# Use of duplex mutation primers for real-time PCR quantification of hepatitis C virus RNA in serum

**Published:** 2011-07-01

**Authors:** Qian-Feng Xia, Yang-An Wen, Ping Liu, Pu Li, Jin-Bo Liu, Xi Qin, Shi-Yun Qian, Zhi-Guang Tu

**Affiliations:** 1 The Key Laboratory of Laboratory Medical Diagnostics, Ministry of Education, the Faculty of Laboratory Medicine, Chongqing Medical University, Chongqing, China; 2 The Faculty of Laboratory Medicine and Tropical Medicine, Hainan Medical College, Haikou, China

**Keywords:** HCV, Quantification, PCR, Duplex mutation primers, Hepatitis

## Abstract

**Background:**

The duplex mutation primers offer many advantages over other multi-labeled probes for real-time detection of amplification products.

**Objectives:**

To develop and validate a novel real-time PCR for quantification of HCV RNA based on the duplex mutation primers technology.

**Materials and Methods:**

The duplex mutation primers were selected in the highly conservative 5' non-coding region (5'NCR) of the HCV RNA. The assay was validated with the Viral Quality Control panel, which also includes Chinese HCV RNA standards.

**Results:**

The detection limit was 57 IU/mL, and a good linear correlation in the range of 102-108 IU/mL was revealed (r(2) = 0.999) with the novel method. This assay has a dynamic range of at least 8 log10 without the need for specimen dilution, good clinical intra- and inter-run precision, and excellent correlation with a commercially available assay(r(2) = 0.95).

**Conclusions:**

The high sensitivity, wide linear range, and good reproducibility, combined with low cost, make this novel quantitative HCV real-time PCR assay particularly well suited for application to clinical and epidemiological studies.

## 1. Background

Quantitative hepatitis C virus (HCV) RNA determinations are currently the standard of care for diagnosis of acute and chronic HCV infections and monitoring for therapeutic efficacy. Several home-made and commercial real-time PCR assays have been used to quantify the level of HCV RNA in serum samples [1][2][3]. The nonspecific dsDNA binding dyes methods are prone to "false positives" in that undesired dsDNA such as primer dimers and other spurious amplicons can bind dyes to produce fluorescence [4]. Well-known specific multi-labeled FRET probes examples include TaqMan [5], molecular beacons [6], scorpion primers [7], and amplifluor [8]. The synthesis and purification of these multi-labeled probes are often either trivial or expensive relative to the pair of fluorophore and the quencher in the same oligonucleotide. Although hybridization probes [9], displacing probes [10] and Q-PNA primers [11] have some advantages over multi-labeled probes. However, both hybridization probes and displacing probes need the phosphatation to block the amplification, are not real single-labeled probes and the costs are increased. We reported a new method based on duplex mutation primers, in which a primer with a fluorophore is attached at its 5'-end, and a complementary oligonucleotide with a single-base mismatched is labeled by a quencher at 3'-end [12]. Incorporation of the fluorophore primer into a double stranded amplicon causes the physical separation of the fluorophore and quencher moieties, and produces an increase of the fluorescence signal, which is the direct indicator of the template concentration. The duplex mutation primers are much more specific than double-strand DNA dyes like SYBR Green I, etc. Unlike other probes [5][6][7][8], duplex mutation primers do not require the double labeled synthesis of fluorophore and quencher on the same molecule, which makes the synthesis of the FRET probe less costly and design more convenient.

## 2. Objectives

The purpose of this study was to report the development of a quantitative real-time PCR HCV RNA assay using duplex mutation primers technology. We evaluated the sensitivity, specificity and efficacy of this assay and its usefulness for the diagnosis of HCV infection. The correlation between results with this assay and with the TaqMan assay was investigated.

## 3. Materials and Methods

### 3.1. Patient samples

The clinical samples included 92 sera collected from patients with an acute or chronic HCV infection. To test for specificity, we used samples anonymously obtained from each of the following groups: apparently healthy volunteers of our hospital (n=60), HBV-monoinfected patients (n=45), and HAV-monoinfected patients (n=5). All healthy volunteers had normal serum ALT levels and were negative for antibodies to HCV by ELISA. For all samples, sera was separated from whole blood, aliquoted, and stored at -20°C until it was further processed.

### 3.2. Viruses for analytical studies

The Chinese National standard for HCV, which has an assigned concentration of 1.25×10(6) IU/mL, was serially diluted in negative human plasma to a final concentration ranging from 1×10(6) to 1×10(2) IU/mL, and were used as reference standards for each real-time PCR run. Analytical verification studies (measurable range, LOD, and precision) were performed with a commercial panel of clinical specimens containing HCV Gt 1a, Gt 2b, and Gt 3a viruses (DaAn, Ltd. Guangzhou, China). For a standardized evaluation, we obtained a Chinese National reference viral quality control (VQC) plasma preparation panel containing well-characterized HCV RNA levels. These samples were tested extensively and contain HCV RNA levels ranging from no HCV RNA to 107 HCV molecules per mL.

### 3.3. Nucleic acid extraction

Total RNA was extracted from 200 µL of serum with a Trizol kit (Invitrogen, USA) according to the manufacturer's instructions. After the extraction by the Trizol kit, the total amount of extracted nucleic acids was solved in 100 µL.

### 3.4. Reverse transcription

A total volume of 25 µL of reverse transcription reaction mixture was prepared as follow: 1 µL of random primers (0.2 µg/µL, Promega, USA) was added to 5 µL of extracted HCV RNA. The mixture was denatured at 70°C for 5 minutes and cooled on ice immediately. Then 5 µL of 5× reverse transcription buffer, 0.75 µL of RNasin (40 U/µL, Promega, USA), 1 µL of M-MLV reverse transcriptase (200 U/µL, Promega, USA), 1.25 µL of dNTP (10 mM, Promega, USA) and 11 µL of RNase-free water were added into the reaction tube. The tube was flicked gently and incubated for 60 minutes at 37°C. The reaction was terminated at 95°C for 5 minutes. Reverse transcription products (cDNA) were frozen at -20°C until further use. The presence of HCV cDNA in these samples was then analyzed with commercial TaqMan kit (Da'an Ltd., Guangzhou, China) and our method.

### 3.5. Preparation of duplex mutation primers

The fluorophore primers (5'-FAM-CGACACTCCACCATAGATCAC-3') and the reverse primers (5'- AGGCTGCACGACACTCATAC -3') were used to amplify a 94-bp fragment in the highly conservative 5' non-coding region (5'NCR) of the HCV RNA. The fluorophore primers were quenched by partly complementary oligonucleotides (5'-GTGATCTATGGCGGAGTGTCG-Dabcyl-3') of a single-base mismatched labeled with a quencher at 3'-end. All of the primers were selected using the primer premier 5.0 software and were synthesized, purified and labeled by Takara Ltd. Dalian, China.

### 3.6. Real-time PCR system

Real-time PCR was performed with a Rotor-Gene 3000 (Corbett Robotics, Australia). The total volume of the real-time PCR was 20 µL containing 5 µL of cDNA, 4 µL duplex mutation primers (2.0 µM of the fluorophore primer, 2.0 µM of the quencher strand), 4 µL reverse primer (2.0 µM), 2.5 µL each dNTP (1.6 mM, Takara Ltd. Dalian, China), 2 µL 10× buffer, 0.2 µL of Taq polymerase (5 U, Takara Ltd. Dalian, China) and 1 µL MgCl2 (25 mM, Takara Ltd. Dalian, China), adding distilled water to 20 µL. The cycling parameters were an initial denaturation at 94 °C for 5 min, 40 cycles of 93 °C for 15 s, and 48 °C for 15 s. The signal detection was preformed at 48 °C during each cycle. To quantify the starting template in samples, the samples were run in along with a standard curve generated from 10-fold serially diluted standard serum of a known concentration.

### 3.7. Lower limit of detection

To test for the lower limit of detection, the stock of the commercial panel of clinical specimen was diluted with sterile water to 400, 200, 100, 50, 25, 12.5, 6.3, 3.1, and 1.6 IU/mL. Eight replicates of each dilution and two negative controls were then tested by our method.

### 3.8. Linearity

To test the linearity of the quantitative PCR, the stock of the HCV reference standard at a concentration of 1.25×10(6) IU/mL, was diluted stepwise (1:10), and four to six replicates were tested per dilution. Furthermore, a high concentration plasma sample (as measured by the HCV Diagnostic kit) from a patient was diluted stepwise (1:10), and two replicates per dilution were tested.

### 3.9. Precision

To test the inter-run precision, four samples of HCV RNA from patients with low, medium and high levels of viremia as well as one sample from healthy volunteers were aliquoted and stored at -20 °C before use. These five samples were repeatedly assayed five times in five independent experiments. The results of quantitation, along with standard deviations and coefficients of variation (CVs), intra-run precision were determined by measuring the amount of HCV RNA in the 5 samples mentioned above within one PCR run.

### 3.10. Correlation between the novel PCR assay and a commercially assay

The HCV RNA real-time PCR assay was compared with a commercially available TaqMan assay (Diagnostic kit for HCV, DaAn, China) to determine its usefulness for a clinical virology laboratory. A total of 202 serum specimens were tested by both methods. Samples were not tested simultaneously on both platforms but were first tested by the commercially available assay and then frozen at -20 °C until RNA extraction and the novel PCR testing. Specimens throughout the range of quantification were chosen for analysis. Data were logarithmically transformed before analysis.

### 3.11. Data analysis

Reproducibility was estimated by computing CVs. The difference of the quantitative results between the commercial TaqMan kit and the developed method was assessed by the paired-sample Student's t test or the Sign test when the results were not distributed normally. Probit statistics was performed to determine the limit of detection of our HCV real-time PCR assay. P < 0.05 were considered statistically significant.

## 4. Results

### 4.1. Real-time PCR detection

**Figure 1:**
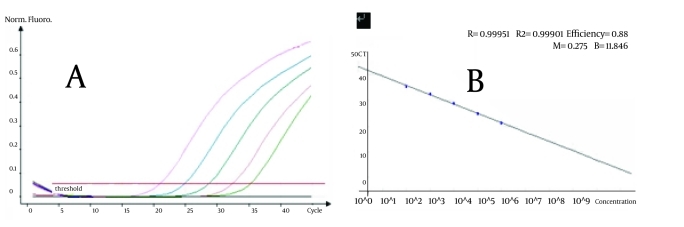
Fluorescent curves of the standard dilution series.

The results of real-time PCR analysis of the single copy conservative sequence in HCV genome as a function of template copy number using our primers are shown in Figure 1. A Chinese National standard for HCV dilution series of 10(2)-10(6) IU/mL was amplified in each PCR run. The cycle threshold (Ct) was automatically set by the instrument software. The calibration curve of Ct vs. template copy number showed linear regression analysis of the best-fit line yielded a good coefficient of determination (r(2)) of 0.999 (Figure 1). Analysis of reaction products by electrophoresis revealed that the correct size of the reactions produced amplicons.

### 4.2. Limit of detection

The samples yielded positive results in 100% of replicate reactions at concentrations greater than or equal to 100 IU/mL. Below the concentration, the detection failure rate increased as the concentration decreased (Table 1). A clinical sample was quantified by serially diluting the stock solution, performing eight amplifications with the novel method, and calculating the concentration from the fraction of negative reactions by application of Poisson's law. A detailed probit analysis indicated that the limit of detection (the concentration at which 95% or more of the replicates tested gave a positive response) of the test was 57 IU/mL.

**Table 1:** Limit of the HCV RNA real-time PCR assay

**Concentration**, IU/mL	**positive**, % (positive/total replicates, No.)
**400**	100 (8/8)
**200**	100 (8/8)
**100**	100 (8/8)
**50**	87.5 (7/8)
**25**	50 (4/8)
**12.5**	37.5 (3/8)
**6.3**	12.5 (1/8)
**3.1**	0
**1.6**	0

### 4.3. Linearity

Linearity was confirmed using a patient sample with high levels of HCV RNA. After various dilutions, the nucleic acid was extracted and the HCV RNA was quantified. Linearity was observed from 10(2) up to 10(8) IU/mL in the sample.

### 4.4. Precision

The inter- and intra-run variability were tested (see Materials and Methods); the results are shown in Table 2.

**Table 2:** Study of intra and inter-run variability of the HCV RNA real-time PCR assay to determine its precision

	**HCV RNA** , IU/mL					**Mean**	**SD**	**CV**, %
	**1**	**2**	**3**	**4**	**5**			
**Intra-run**								
**1**	2.14×102	2.36×102	2.24×102	2.01×102	1.95×102	2.14×102	1.67×101	7.80
**2**	1.39×103	1.21×103	1.24×103	1.31×103	1.19×103	1.27×103	8.2×101	6.46
**3**	5.17×104	5.36×104	5.25×104	5.19×104	5.12×104	5.22×104	9.2×102	1.76
**4**	7.45×107	7.37×107	7.39×107	7.41×107	7.31×107	7.39×107	5.2×105	0.70
**Control**	N a	N	N	N	N	-	-	-
**Inter-run**								
**1**	2.44×102	2.31×102	2.12×102	2.19×102	1.85×102	2.18×102	2.22×101	10.18
**2**	1.39×103	1.32×103	1.20×103	1.27×103	1.15×103	1.27×103	9.50×101	7.48
**3**	5.17×104	5.24×104	5.09×104	5.31×104	5.37×104	5.24×104	1.11×103	2.12
**4**	7.51×107	7.37×107	7.30×107	7.21×107	7.47×107	7.37×107	1.23×105	1.67
**Control**	N	N	N	N	N	-	-	-

^a^ N: negative

### 4.5. Specificity

To determine the specificity of the real-time PCR detection assay, we used 110 samples anonymously obtained from each of the following groups: apparently healthy volunteers of our hospital (n=60), HBV-monoinfected patients (n=45), and HAV-monoinfected patients (n=5). None of the samples showed false-positive reactions in duplicate.

### 4.6. Comparison between two different quantification assays

**Figure 2:**
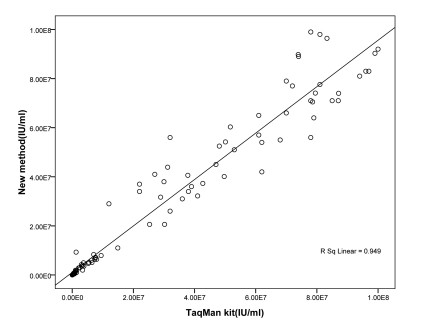
Graph shin correlation of the HCV real-time PCR ass av results with the new method results for 92 HCV-positive clinical spcimens (numerical values e not listed). Thre was excellent correlation (r 0. 95) between the two assays for all samples.

The presence or quantity of HCV RNA in the sera of the 92 HCV patients was determined by our method and commercial TaqMan kit separately. There was no significant difference among the tested results with two methods in 92 HCV hepatitis patients (P = 0.723). A significant positive linear correlation was found (r(2) = 0.95, Figure 2).

## 5. Discussion

Methods for HCV RNA quantitation must be specific, sensitive, reproducible, and accurate. Analysis of large number of samples requires a rapid and manageable protocol that minimizes as much as possible post-PCR manipulations. Endpoint PCR-based techniques for HCV RNA have shown a limited dynamic range, usually within 4 logs, affecting the quantification of moderate to high levels of viremia [13]. Real-time PCR is designed to quantify nucleic acids in the exponential phase of the reaction, allowing for a more reliable and precise quantification over a much wider dynamic range. Quantitative tests of HCV RNA levels in serum are currently the standard of care for the diagnosis of acute and chronic HCV infections and monitoring for therapeutic efficacy [14]. In this study, we described the development and validation of a novel quantitative real-time PCR assay that showed good performance characteristics in terms of the limit of detection, linearity, reproducibility, and specificity. This assay has good clinical sensitivity at 57 IU/mL using an initial sample volume of only 200 µL, at least an 8 log10 linear dynamic range without the need for specimen dilution. This linearity was well in line with the linearity of other assays performed on the Cobas TaqMan HCV platform [15]. The low variability of this assay makes a twofold decrease or increase in the serum HCV RNA level already significant. The mandatory requirement of 100% specificity was achieved, as none of the controls, including healthy donors and patients infected with HBV or HAV, was tested positive HCV RNA. Nevertheless, the number of control samples need to be increased to further confirm these findings. The high reproducibility and linearity of the assay described proved to be comparable with one commercially available assay. Our method has several advantages over the other multi-labeled probes methods; higher specificity: the primers are duplex form and cannot hybridize with each other at lower temperature than their melting temperature that minimizes non-specific annealing in the course of amplification. Higher sensitivity: after primer-target hybridization, the distance between the fluorophore and the quencher changes from close proximity to totally free separation. This property endows them with stronger signal than other internal FRET probes. Higher cost/efficiency: single-labeled oligonucleotides structure makes the synthesis less costly and design more convenient than multi-labeled probes. In conclusion, a duplex mutation primers real-time PCR system with high cost/efficiency, rapid, sensitive and high specificity has been developed for the quantitative detection of HCV RNA. The assay is suitable to be used for both clinical and epidemiological studies.
